# Design and Simulation of a Hierarchical Parallel Distributed Processing Model for Orientation Selection Based on Primary Visual Cortex

**DOI:** 10.3390/biomimetics8030314

**Published:** 2023-07-16

**Authors:** Hui Wei, Jingyong Ye, Jiaqi Li, Yun Wang

**Affiliations:** Laboratory of Algorithms for Cognitive Models, School of Computer Science, Fudan University, Shanghai 200082, China

**Keywords:** brain-like model, retina, orientation selection, programmable devices, primary visual cortex

## Abstract

The study of the human visual system not only helps to understand the mechanism of the visual system but also helps to develop visual aid systems to help the visually impaired. As the systematic study of neural signal processing mechanisms in early biological vision continues, the hierarchical structure of the visual system is gradually being dissected, bringing the possibility of building brain-like computational models from a bionic perspective. In this paper, we follow the objective facts of neurobiology and propose a parallel distributed processing computational model of primary visual cortex orientation selection with reference to the complex process of visual signal processing and transmission between the retina to the primary visual cortex, the hierarchical receptive field structure between cells in each layer, and the very fine-grained parallel distributed characteristics of cortical visual computation, which allow for high speed and efficiency. We approach the design from a brain-like chip perspective, map our network model on the field programmable gate array (FPGA), and perform simulation experiments. The results verify the possibility of implementing our proposed model with programmable devices, which can be applied to small wearable devices with low power consumption and low latency.

## 1. Introduction

Advanced primates have evolved over a long time to optimize powerful and well-developed visual systems. The human visual system, as a sophisticated and complex information acquisition and perception system, provides a solid foundation for people to accomplish tasks such as awareness, cognition, and understanding. However, visual impairment and lesions cause great inconvenience to people with visual impairment. Some diseases, such as glaucoma, are caused by the axonal death of ganglion cells in the retina, resulting in impaired visual fields. Macular degeneration is caused by the degeneration of retinal pigment epithelial cells, leading to the degeneration and death of photoreceptors. Due to the insufficient bionics, no major practical application breakthroughs have emerged from the prospect of applications in biomedical engineering and functional rehabilitation. On the one hand, the lack of understanding of brain-coding mechanisms leads to uncoordinated human–machine interfaces. On the other hand, the implementation of previous computational models usually requires a large amount of hardware resources, ignoring the need for portability and low power consumption. For example, the impaired need functional assistive devices respond instantly and reliably with a low cost, which places high demands on portability and computational efficiency. We hope to design wearable brain-like visual aids device to solve similar problems, and it is a good choice to develop tools from an embedded perspective in terms of hardware implementation [1].

In terms of approaches to solving visual tasks, we prefer to study the processing of objects in the human visual system from the direction of bio-vision theory rather than computer vision theory because it is supported by more direct and reliable biological arguments and has a certain interpretability and robustness. Although computational models utilizing deep learning have emerged and shown good accuracy, they also suffer from network structures that do not facilitate understanding, are vulnerable to attacks [2,3], and generate high power consumption [4,5]. Bio-vision systems solve all these problems extremely well and achieve an excellent balance between real-time processing, accuracy, robustness, and energy consumption. At the same time, the biological mechanism can effectively simulate the function of visual structures, which helps make designs that more easily achieve information transfer with the brainand has broad application prospects in the fields of biomedical engineering.

One way of researching brain-like computing is to study how the brain’s structure is adapted to its functional needs. Some researchers have constructed brain-like computing models based on certain parts of the brain structure and neurocomputational principles. For example, Salamat et al. proposed a brain-like unsupervised clustering method based on hyperdimensional computing, which maps low-dimensional data to high-dimensional data for processing clusters [6]. Wei et al. proposed computational models that simulate the structure of functional columns of the visual cortex and nonclassical receptive fields [7,8,9,10]. These studies show how to design computational models from a neuromorphic perspective with the potential of in-memory computing.

Tanaka et al. proposed a brain-like learning model based on the amygdala, which was implemented with hardware and used in a robot [11,12]. Based on the spatial and memory functions of the hippocampus and its spatial navigation function, Aggarwal et al. developed a mathematical model of hippocampal structure, and then implemented the model on the circuit [13]. Cho et al. modeled the behavior of simple cells of the visual cortex by using Gabor functions and implemented the mapping in hardware [14].

Brain-like computing is moving toward the goals of high performance, high parallelism, and low power consumption. These advantages are difficult to achieve with current traditional computing architectures. Designing dedicated architectures and conducting research from the perspective of in-memory computing facilitates the achievement of these goals. The development of storage-based systems has led to an increase in the efficiency of in-memory computing, providing the conditions for reducing hardware resources, on which biologically inspired computing can be used for the purpose of scaling the devices down [15].

We design specific architecture for our computational models on FPGAs. FPGAs are highly customizable and configurable devices that can be customized to better fit the circuit structure of the target system, providing extreme flexibility and reconfigurability for the hardware acceleration of software algorithms. Designing such dedicated architectures can effectively reduce memory bottlenecks, improve overall system computational efficiency, and reduce hardware cost and power consumption.

There has been a great deal of practice in implementing many different kinds of neural networks with FPGAs. Several studies have successfully mapped convolutional neural networks onto FPGAs, exhibiting minimal loss of accuracy while achieving significant improvements in speedup ratios and energy efficiency [16,17]. Some studies implement spiking neural networks on FPGAs and realize model acceleration, indicating that FPGAs are suitable for large-scale cortical simulations [18,19,20]. However, there has been relatively little research into using FPGAs to implement non-traditional multi-level neurocomputational models that strictly mimic visual neurobiological mechanisms and follow visual conduction pathways, which is not just a hardware acceleration of multilayer feedforward networks.

With the continuous development of physiology and anatomy, the principles of the human visual system have been roughly explained from the neuronal level. Some previous research studies build and implement visual computational models on circuits [21,22,23,24,25]. These studies are either limited to individual neural connections or information pathways, while ignoring the overall representation of the early visual system. Based on a large number of physiological and anatomical experiments, there is a certain understanding of the functional hierarchical division of the primary visual cortex, which gives us the possibility to build a visual model based on fundamental physiological knowledge. We simulate these physiological structures and model networks with orientation selectivity. If these orientation signals are transmitted to the visual nerve, it will greatly facilitate the construction of the visual repair system.

This study integrates multiple bottom–up visual pathways in the visual system from the retina, to the lateral geniculate nucleus (LGN), to the visual cortex and proposes a bionic hierarchical network to approximate the process of object formation representation in the visual system, which can also lay a foundation for subsequent higher-level visual tasks. In addition, circuit-based design took a long time to develop and was also expensive to develop and modify according to changes of models. Our proposed FPGA-based network model mimicking the visual system has a multi-layered structure based on both the physiological structure and signal-processing mechanisms, and it follows the anatomical and biological evidence of human visual neural mechanisms more closely. Based on this model, we generate cortical orientation maps that are surprisingly similar to the actual cortical maps and functionally have orientation selectivity.

In researching visual impairment aid devices, there are a variety of research studies that build assistive devices from different perspectives. For example, some studies use sensors, such as ultrasound and laser to locate and distance objects [26,27,28]. Some studies use cameras to acquire images and run computer vision algorithms to calculate obstacle data [29]. There are also papers that use positioning technologies, such as GPS, to build a complete set of hardware wearable devices for the visually impaired [30].

However, the implementation of biological mechanisms is always based on larger hardware resource, ignoring the need for portability and low power consumption. From the perspective of embedded, it is more appropriate to use FPGA as the implementation device for developing wearable devices. Wearable embedded devices are ideal form for medical or assistive devices, which further increase the requirements for size, quality and energy consumption. It needs to be worn on the body and carried around, where portability and durability are necessary. In addition, with the optimization of software algorithms and functional changes, the programmability of the device is also necessary. Multilayer network model design on FPGAs can gain advantages in these aspects. In terms of performance per watt, FPGAs can achieve relatively low energy consumption, which gives longer endurance to portable devices.

In order to find an explainable image representation model and image-processing method, we explore the brain-like mechanism and make the following contributions:

1. We developed a bionic vision model that simulates the process from the retina to the primary visual cortex, which is capable of representing images and giving a neuroscientific explanation of this process.
2. We meticulously mapped the visual pathway model onto FPGAs, effectively integrating biological cell functions with hardware features to achieve parallel distributed neural computation.
3. We performed hardware simulations and parallelism experiments, and the results show that it outperforms the implementation on the central processing unit (CPU) and graphics processing unit (GPU) in terms of parallelism, latency and power consumption.

## 2. Background

### 2.1. Early Visual System

In the human visual system, the visual signals received by the human eye pass through the retina, the LGN, the primary visual cortex, and the upper layers of the higher visual system. Each of these layers has a complex physiological structure and unique visual functions for information processing. The retina is responsible for receiving, sampling, and converting incident light stimuli into bioelectrical signals; the LGN is responsible for collecting the information processed by the lower layers and transmitting it to the upper layers, acting as a relay; and the primary visual cortex and the higher visual cortex are responsible for extracting and recognizing representation information.

#### 2.1.1. Retina

The retina is a cellular layer at the posterior side of the eye, mainly composed of photoreceptor cells, horizontal cells, bipolar cells, anaglyph cells, and ganglion cells (GCs). The photoreceptor cell layer mainly consists of cone cells, which are sensitive to strong light stimuli and have the ability to distinguish colors, and rod cells, which are sensitive only to low light. The horizontal cell layer consists of axon and non-axon horizontal cells, the former receiving input from red-sensitive and green-sensitive cone cells and rod cells, and the latter receiving input from blue-sensitive cone cells, and initially forming the receptive field. The bipolar and anaglyph cells sort and converge on the transmitted information to obtain different signals of high visual acuity in light and dark conditions. The GCs are the last and most functionally important processing layer in the retina. And several layers of cells in front of them form the receptive field, which collects signals for the ganglion. It mainly consist of magno (M) cells and parvo (P) cells. M cells are sensitive to small differences in light intensity, and P cells are sensitive to color contrasts.

#### 2.1.2. Lateral Geniculate Nucleus

The LGN, a visual information processing structure in the thalamic receptive nucleus, consists mainly of the parvocellular and magnocelluar layers, which have the same receptive fields as the GCs and both receive projections from the retinal nasal and temporal GCs, and are very important signal caches and relay stations.

### 2.2. Visual Cortex

The visual cortex is distributed in both the left and right brain and performs advanced processing of information transmitted from lower layers.

#### 2.2.1. Primary Visual Cortex

The primary visual cortex, also known as Brodmann’s area 17, is a well-studied area that receives information from the LGN layer and starts the primary processing of visual information. The primary visual cortex is composed of six layers, with different divisions of labor among the layers, mainly consisting of simple and complex cells. Simple cells are mainly distributed in area 17, layer 4, which has a small receptive field and does not respond to diffuse light over a large area, while they have a strong response to bar stimuli in a certain direction at the edge of the receptive field [31]. Complex cells are mainly located in area 17 and area 18. In contrast to simple cells, they have certain requirements for their length in order to respond to strip stimuli. The different reactions of various cells are abstracted to give rise to the concepts of the cortical functional column, the ocular dominance column, etc.

#### 2.2.2. Other Visual Cortex

In addition to the primary visual cortex, there are also visual cortices, such as V2, V3, V4, and V5 (MT), which are in the upper layers of the primary visual cortex. V2 receives information from V1 and gives strong feedback to it, and continues to pass it upward, V4 is sensitive to higher geometric shapes [32], and V3 and V5 play an important role in motion perception [33].

## 3. Methods

We focus on this existing knowledge in anatomical structure and information processing functions and use it as basic constraints for brain-like computational model design. We develop a hierarchical network model of visual pathways based on neurobiological mechanisms. Each layer of the network architecture is an abstract model of a particular function of the visual system. Also, we conduct some experiments to verify the feasibility of model.

### 3.1. Hierarchical Network Computational Model

By studying the physiological structure and function of the retina, LGN, and primary cortex, it can be found that primary visual cortex cells have orientation selectivity, which is important for object contour extraction and representation. In order to simulate the information processing mechanism of human vision, we abstract the main cellular structures in the physiological visual system and establish an orientation-selective model of the early visual system.

**Figure 1:**
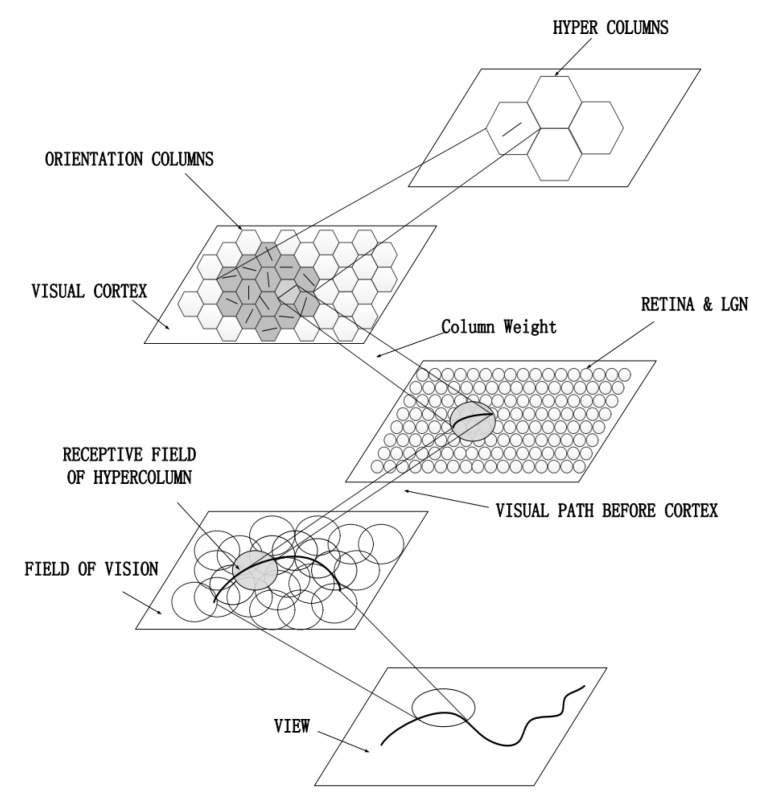
Primary visual cortex orientation selection hierarchical network. The input of the model is a natural image. Processing units include the receptive field layer, retinal and LGN layers, and primary visual cortex, and each layer of the computational model simulates the processing of images by cells in a layer of the early visual system.

The model is shown in Figure 1. The receptive field layer simulates the oculomotor scanning process of the human eye and simulates some cells in the retina to segment the image in the receptive field into separate receptive fields; the retinal and LGN layers carry out the difference-of-Gaussians (DOG) processing of pixels in the receptive fields as a GC model, and the primary visual cortex carries out the orientation processing of the results of the upper layer processing as an orientation column model to present the representational information and form a cortical orientation map as the output.

### 3.2. Modeling of Ganglion Cells

When we view an image, the visual information within a certain field of vision enters the photoreceptor cells as the eye turns. The visual information is gradually processed through the retinal layer to form the concentric receptive field. Bipolar cells generate graded potentials from information in the receptive field and transmit them to ganglion cells. The receptive field formed during this process has a central peripheral antagonistic mechanism [34]. It is impossible to create a negative firing frequency; the retina splits its information pathways to OFF and ON to encode both positive and negative derivatives. For the on-center and off-center areas of the receptive field, when both are stimulated with the same degree of light intensity, a bipolar cell shows no significant response to it, and the output of this cell to the upper layer is almost zero. A bipolar cell responds significantly to the stimulus only when the contrast between the stimuli in the two areas is greater. And the DOG model [35] can simulate the physiological properties of this receptive field very well. Based on the two-dimensional DOG model, the output values of the cells in receptive field at position ($x_{0}$,$y_{0}$) are determined by a combination of excitation and inhibition input photoreceptor cells at ($x_{i}$,$y_{i}$). And the following simulation function is used to represent it:

(1) $$R\left(x_{0},y_{0},\sigma \right)=\sum_{i=0}^{n}p\left(x_{i},y_{i}\right)\times \frac{1}{\sqrt{2\pi }\sigma e^{-\frac{{\left(x_{i}-x_{0}\right)}^{2}+{\left(y_{i}-y_{0}\right)}^{2}}{2\sigma^{2}}}}$$

where σ denotes the parameter of the Gaussian function, *R* is the output of cells in the receptive field, *x*, *y* are the relative positions of cells, and p(x,y) is the output of photoreceptor cells in relative position (x,y).

Since on-center parvo cells (On-P) and off-center parvo cells (Off-P) make up approximately 90% of GCs, we mainly model these two types of cells. The response function is as follows:

(2) $$GC_{on}\left(x_{0},y_{0}\right)=R\left(x_{0},y_{0},\sigma_{cen-on}\right)-R\left(x_{0},y_{0},\sigma_{sur-on}\right)$$

(3) $$GC_{off}\left(x_{0},y_{0}\right)=R\left(x_{0},y_{0},\sigma_{cen-off}\right)-R\left(x_{0},y_{0},\sigma_{sur-off}\right)$$

where $\sigma_{cen-on}$, $\sigma_{sur-on}$, $\sigma_{cen-off}$, $\sigma_{sur-off}$ are the parameters of central and peripheral receptive field of On-P and Off-P.

The GCs then process the received information and perform selective output, which in physiology is the process of converting graded potentials into action potentials. GCs are also the first cells to emit action potentials during information processing. Only when the signal strength is greater than its own threshold potential value does the GC generate an action potential to transmit the information backward. According to the relationship between the resting potential, threshold potential, and peak action potential of the cell (the resting potential is approximately −70 mv, while the potential causing the opening of sodium channels is approximately −50 mv and the action potential peak is approximately +35 mv), we set the threshold value of the model to 0.2:

(4) $$Threshlod=(GC_{max}-GC_{min})\times \alpha$$

where $GC_{max}$, $GC_{min}$, and α are the maximum and minimum of the GC responses, and a hyperparameter related to the type of GC.

### 3.3. Modeling of Orientation Columns in the Primary Visual Cortex

When visual information is transmitted to the visual cortex via the LGN, a variety of cells in the primary visual cortex are activated to varying degrees to form an initial orientation representation of the object. According to biological discoveries, cortical cells are orientation selective and arranged in a specific structural manner. Cortical puncture experiments showed that when microelectrodes are inserted perpendicular to the surface of the visual cortex, the receptive fields of various cells are found to be mostly overlapping, and the preferred optimal orientation is similar. When microelectrodes are inserted in an approximately horizontal orientation to the surface, the orientation selectivity of the cells changes continuously.

Subsequently, the cortical ice block model [36] was proposed to simulate two functional structures of the cortex, the ocular dominance column and the orientation column. The former indicates which eye is more likely to influence visual processing, while the latter detects orientation features. We use the orientation column as the main bionic and computational modeling object in this study.

**Figure 2:**
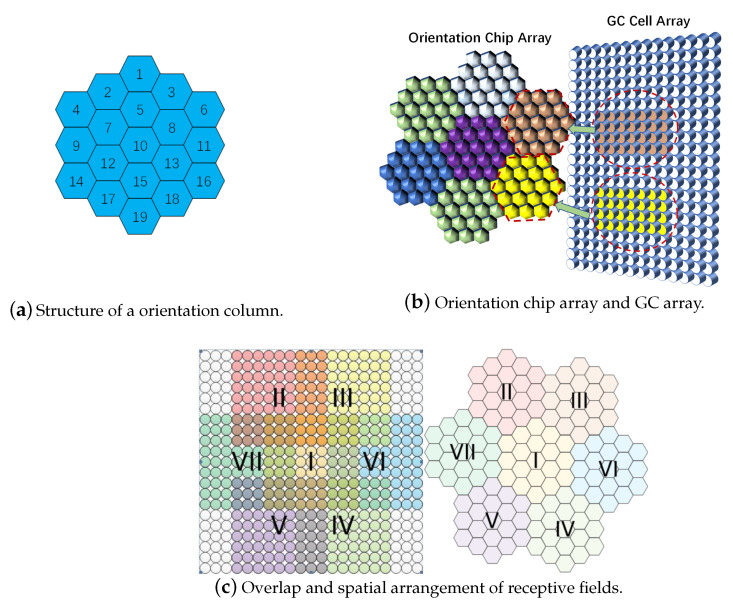
Orientation columns. (**a**) The logical structure of a orientation column. (**b**) Orientation chip array will receive input from GC cell array within receptive field. (**c**) In terms of their range of receptive fields, the mutual receptive fields will overlap. In terms of the logic array, they have a specific, non-overlapping alignment.

We establish an orientation column model to represent the concept of the functional column in the cortex, which is a functional module with orientation selection. It consists of orientation chips and receives processing information from GCs, and the logical structure of a orientation column is shown in Figure 2a. Considering the scalability of the orientation chip and orientation column structure, we design the structure of the orientation chips in such an arrangement shown in Figure 2b. The orientation chips represent different kinds of cells sharing the same receptive field under the same orientation column. Figure 2b expresses the relationship between the GC array and orientation column array as well. The receptive field of the orientation column is composed of receptive fields of all cells in it as shown in Figure 2c.

### 3.4. Training Orientation Columns by SOM

In biological neural networks, neurons have competitive relationships with each other, and such relationships are self-learned by neurons when they compete. There is a clustering effect between neurons.

Often, neurons cluster together to accomplish similar functions, such as functional columns, and changes in neurons simultaneously affect surrounding neurons to varying degrees and produce a lateral inhibitory effect, i.e., they will send activation signals to neurons that are relatively close and inhibition signals to neurons that are relatively far away.

The self-organizing map (SOM) [37], an artificial neural network for multidimensional classification, is an unsupervised learning network that mimics the structural relationships between neurons better than other neural networks. It can both classify the input data effectively and express the topology of the upper layer neural units, and it can represent the competitive yet cooperative relationship between cortical neurons well. In SOM training, when the winning neural node wins, it causes the neural nodes near its topology to receive some of the learning gain, which is extremely consistent with neurobiology.

**Figure 3:**
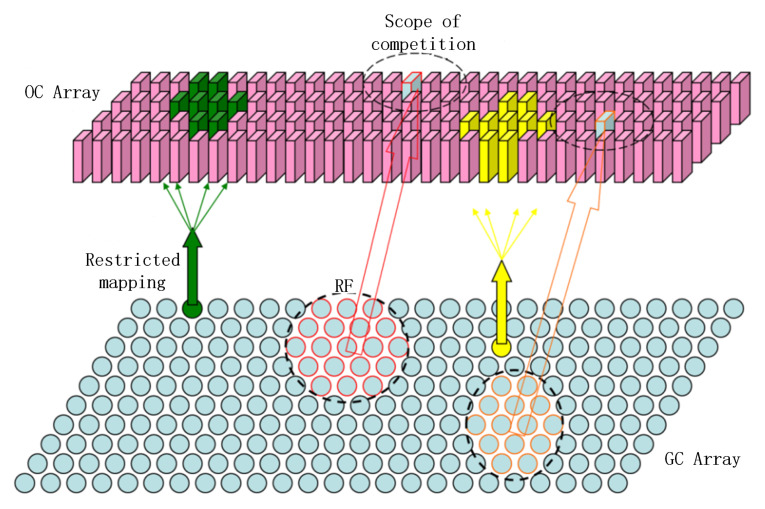
SOM with restricted connectivity. The upper part represents the array of GCs and the lower part represents the array of orientation chips. GCs under the same receptive field are only competed by the orientation chips in the same orientation column, and the same location in the image may be processed by different GCs and subsequently compete with different orientation chips.

Unlike traditional self-competitive networks, our self-competitive model is not fully connected to the lower layer inputs but instead has limited connections. The upper layer neurons compete and learn from the output of lower layer GCs within the same receptive field in terms of orientation columns as shown in Figure 3.

### 3.5. Feasibility Verification Experiment

In order to verify the feasibility of the above hierarchical network computational model, we conduct several image-processing experiments on the model.

**Figure 4:**
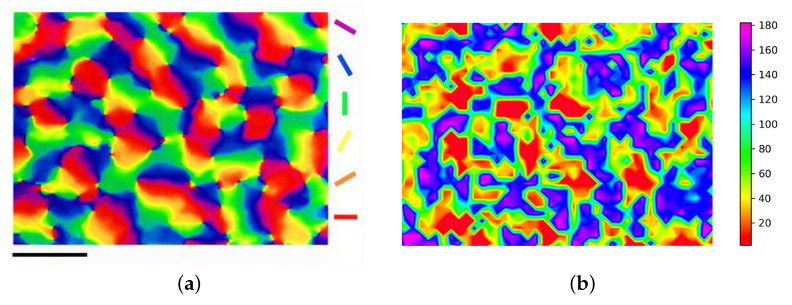
The cortical pinwhee experiment results. (**a**) Stained organisms map [38]. (**b**) Orientation columns map.

Figure 4a shows the map of cortical functional features. The cortical pinwheel is a unique phenomenon in the cortical orientation map in which singularities, i.e., orderly and uniform increases in orientation selectivity, are produced, which is more likely to occur at the adjacent boundaries of multiple orientation columns. The appearance of cortical pinwheels verifies the validity of the model developed. Training by our theoretical model leads to the results shown in Figure 4b. Our results have a high similarity to the orientation map of biological staining with voltage-sensitive dyes. To some extent, this generated cortical orientation map already has some of the functions of a real cortical column. Putting this to good use might be an aid in repairing visual impairment.

**Figure 5:**
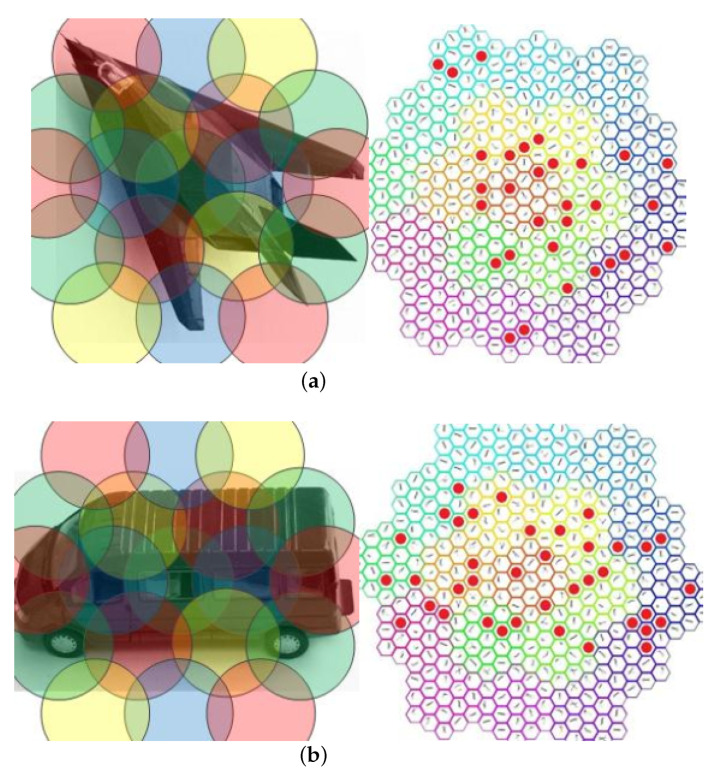
Representation of objects.

In the orientation chip layer, each orientation chip activated by the orientation column is identified, and a roughly characterized pattern of the object is derived. As shown in Figure 5, in this representation mode, the representation results of the same or similar objects are approximately the same. Figure 5a is formed by object rotated by a certain angle, and its representation results are also rotated by a certain angle, which can be seen to have rotation invariance. However, the representation results are very different among different objects and present different distributions on the orientation feature space as shown in Figure 5b.

## 4. FPGA Design of Bionic Vision Model

We explore the advantages and possibilities of implementing the primary visual cortex computing model with FPGAs. Then we propose the FPGA-V1 orientation selection computing model (where V1 refers to the primary visual cortex), design processing modules corresponding to each level of the model, and achieve parallelization and pipelining on it.

### 4.1. Mimick Cortical Computing with FPGA

Many neural computations are implemented through software programming via von Neumann-type computers, whose hardware structures are often fixed and much lower in parallelism granularity than those between biological neural networks. FPGAs are currently faster, more efficient, flexibly configurable, and support non-von Neumann structured designs that can achieve higher processing speeds than CPUs with a high degree of parallelism.

There are many applications using FPGAs, such as in embedded applications, where iterative product updates can be performed quickly due to their programmability, reducing development costs in biomedical applications such as ultrasonic scanning and medical optical imaging, where image reconstruction and image analysis can be performed instantly at a faster rate than software.

Considering the following characteristics, we use FPGAs as the hardware to implement neural computing.

- In the human visual system, there is a huge variety of neurons with different functions, which are distributed in different layers of the visual system and uniquely arranged into cell arrays. There are various kinds of memory types in FPGAs, and storing these cells in FPGAs according to the functional characteristics of the cells is more suitable for simulating the processing and transmission between cell layers compared to a computer, which only uses memory. FPGAs are also more capable of performing large-scale neural computation.
- In the human visual system, neural transmission is accomplished by chemicals or electrical signals at extremely fast speeds. One of the outstanding advantages of FPGAs is the speed of processing information. Unlike CPUs, FPGAs do not need to go through fixed cycle operations, such as fetching, translating, and executing. Furthermore, FPGAs do not require shared memory to maintain consistency, enabling faster data processing calculations and lower data transmission latency.
- In the human visual system, cells are individual entities that collaborate with each other, and this parallel distributed processing should be imitated to achieve multi-level computing. FPGAs are inherently highly parallel and are essentially a complex array of circuit combinations that can well reflect the highly parallel distributed processing characteristics of brain-like computing. Compared to GPU, it can achieve not only data parallelism but also pipeline parallelism.
- The efficiency of neuronal computation transfer is very high, and the energy consumption is very low. FPGAs can achieve the same computational efficiency with lower power consumption than CPU or GPU.
- If we consider the brain as a computer, its power is only nearly 20 W, possessing an extremely excellent energy-consumption ratio. The core of brain-like computing is the “computing-in-memory” structure. To some extent, we can reduce the power consumption of memory access by FPGAs. Moving toward storage and computing integration can significantly improve system efficiency and energy consumption.
- Brain-like bionic mechanisms help us transfer information to the brain. Bionic computational models can be used in a wide range of biomedical engineering applications, such as functional compensation and repair for people with certain visual impairments. It is also a very necessary choice to design an embedded system based on bio-vision and aiming at portable and low-energy image processing modules.

### 4.2. FPGA Architecture for Primary Visual Cortex Computing Model

**Figure 6:**
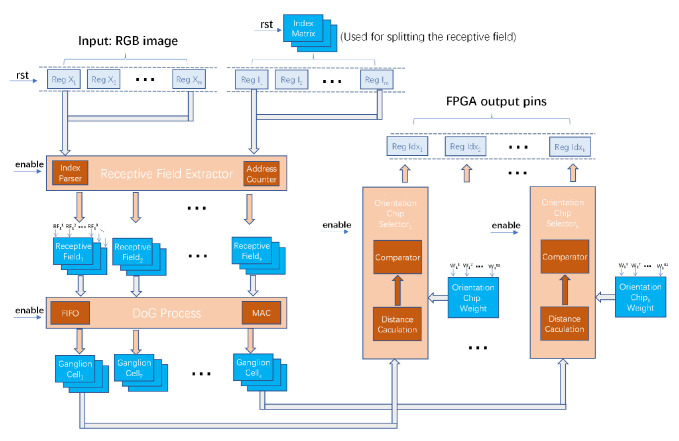
Architecture of orientation selection model. The receptive field extractor is designed to map the computational unit arrays of the simulated retinal and LGN layers. It segments and combines them into a GC receptive field array. The DOG process module, which corresponds to the retinal and LGN layers, performs DOG processing and outputs the results to the orientation chip array. The orientation chip selector, which corresponds to the primary visual cortex, performs orientation chips selection in terms of orientation columns, and then it outputs the index of the activated orientation chips.

In the orientation selection model of the early visual system presented above, we include the receptive field layer, the retinal and LGN layers, and the primary visual cortex. According to the model, we map it to the FPGA architecture in terms of layer. As shown in Figure 6, we use the grayed-out RGB format image as the input. And the output should be a series of indexes activated orientation chips. We expect the processing to be parallelized from the input to the output. Each processing unit is used to correspond to the function of a cellular layer of the visual processing.

#### 4.2.1. Splitting the Receptive Field

In the retinal structure, numerous horizontal cells receive photoreceptor signals and form individual receptive fields through electrical coupling between horizontal cells, which overlap with each other. How can the image be segmented into many receptive fields in the FPGAs so that the pixel data contained in them can be uploaded to a specific computational unit? We form a vector of each field into dynamic random access memory (DRAM) (the size of the receptive field is relatively small, and the number of ganglion cells is large, so using distributed memory storage can save storage resources). We design an index matrix method, which is able to split all the receptive fields by scanning the image only once, and it can cope with the case of discontinuous and irregular receptive fields. In the current overlapping approach, a pixel is shared by up to four receptive fields. Each index matrix stores the information of multiple receptive fields corresponding to a pixel at a certain location, and there are four such matrices.

**Figure 7:**
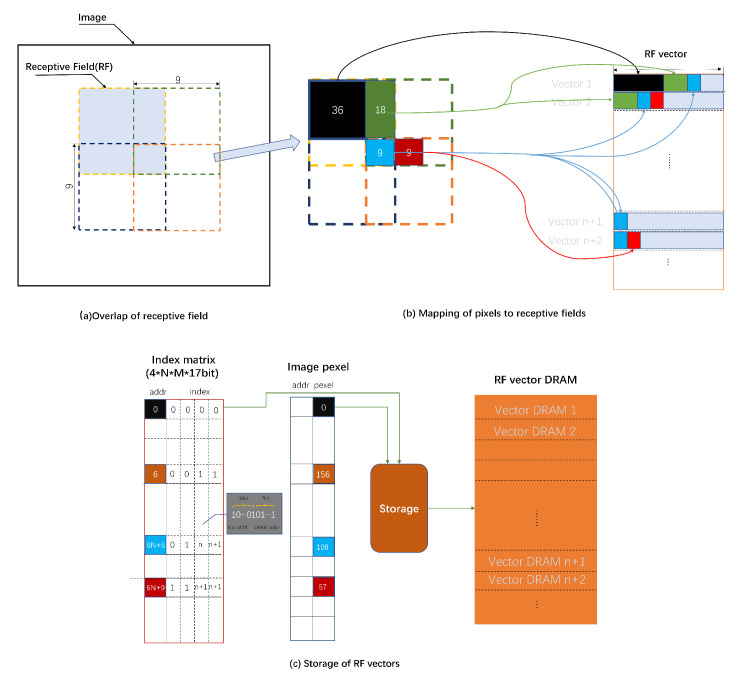
Overlap, segmentation and storage of receptive fields.

**Figure 8:**
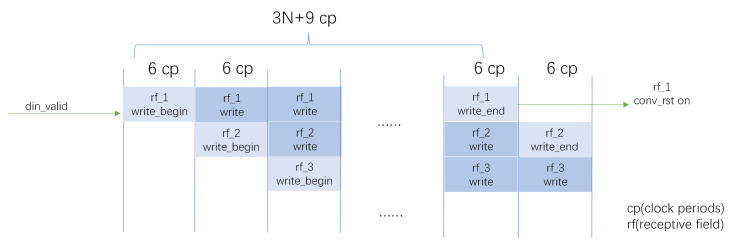
Timing diagram for extracting receptive fields. The writing of pixels to the receptive fields starts after every six clock periods and takes a total of 8 ∗ width + 9 cycles, with each receptive field being written independently.

Figure 7 analyzes the overlap of receptive fields, where a pixel is shared by at most four receptive fields. Figure 7a shows the partitioning of the M × N size image into m × n 9 × 9 size receptive fields. Figure 7b shows the different cases, where the overlapping pixels are shared, e.g., the nine blue pixels are shared by the four surrounding 9 × 9 receptive fields and are stored into receptive field vectors. In Figure 7c, the index matrix is used to record the receptive fields to which the pixel belong, and the indexes are resolved and stored in DRAM in the storage process to facilitate parallelization. The structure of the index is shown as well, where the first 10 bits represent the index of receptive fields, and the last 7 bits represent the offset of the storage location within the block. The index value of each location is generated by the index generation algorithm (Algorithm 1), and the index matrices are pre-written into the FPGA storage. Finally, the pixel values are written to different receptive field DRAMs according to the index values. The writing timing of receptive field is shown in Figure 8. As the input signal is converted to valid, numerous receptive field DRAMs are pipelined to write the pixel values and move to the next process with the convolution signal after the writing is completed.
**Algorithm 1** Index Generation**Require:** width: width of picture
    length: length of picture    nrow: number of receptive field in one row    ncol: number of receptive field in one column**Ensure:** IndexMatrix
 1:list←null 2:map←null 3:sidelen←width/(2∗nrow+1)∗3 4:**for** i=0→nrow **do** 5:    **for** j=0→ncol **do** 6:       cor_base←i∗6∗length+j∗6 7:       **for** a=0→sidelen−1 **do** 8:          **for** b=0→sidelen−1 **do** 9:             list.add(cor_base+length∗a+b)10:          **end for**11:       **end for**12:    **end for**13:**end for**14:**for** i=0→width∗length∗sidelen∗sidelen **do**15:       temp←list[i]16:       map[temp].add(i)17:**end for**18:**return** map

#### 4.2.2. DOG Module

In the previous section, we introduced the DOG computational model for the simulation of the central–peripheral antagonistic mechanism, which is able to mathematically well simulate the processing of input signals by retinal and LGN cells. Then we design a DOG processing module to complete the DOG calculation, in which all pixels in the receptive field are ordered, and the output values of GCs are calculated and flow into the orientation chip selection module.

**Figure 9:**
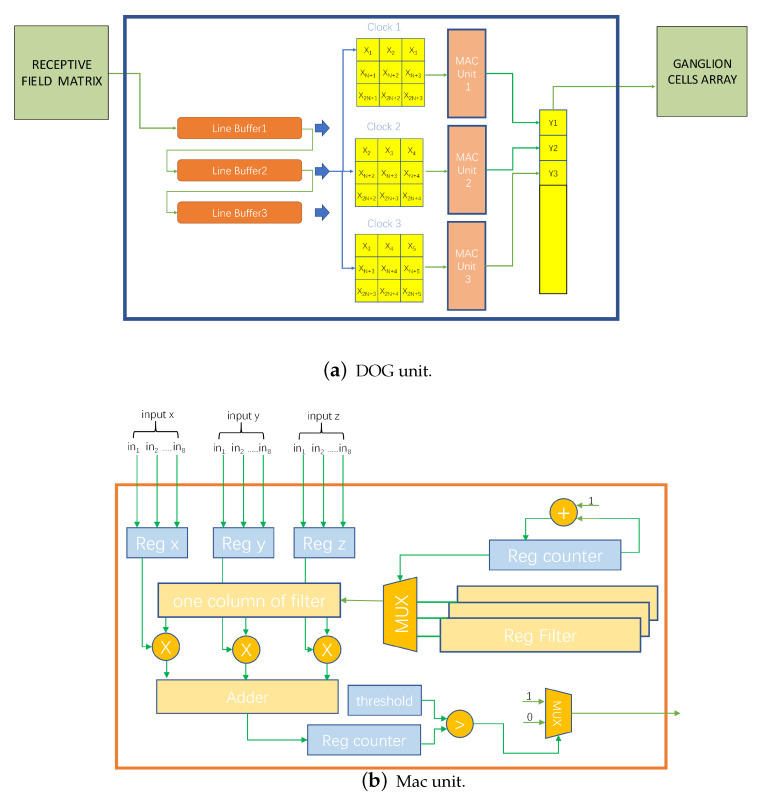
DOG module. Use MAC units parallelly to complete DOG calculations.

We consider the computation of pixels in the receptive field using pipelining to be a convolution process. We set the parameters of the convolution kernel according to the definition of the DOG function, the number of cells, and the size of the receptive field, and perform efficient convolution calculation by using multiple access channel (MAC) units. We store the receptive field in the FPGA in one-dimensional form, and if it needs to be converted to a two-dimensional form for the convolution operation, we need to set up the cache line with the help of first input first output (FIFO) blocks as shown in Figure 9a. We set up three line vectors according to the size of the convolution kernel, we deposit the pixel inflow into the line vectors at any time clock, and we design a signal to indicate when all the line vectors are read in full, which makes the MAC unit start the calculation.

The design of the MAC calculation unit is shown in Figure 9b. The accumulation of the convolutional values of a column is calculated according to different cycles in turn. To improve the computational efficiency, three accumulation units are set up simultaneously for parallel processing according to the size of the convolution kernel, and pipelined computation is performed at different clock intervals. The processed data are thresholded as intermediate nodes, which are binarized and subsequently stored into the GC DRAM array as the final output of the retinal and LGN layers.

#### 4.2.3. Orientation Chip Selector

Based on the primary visual cortex, the orientation columns serve as the basic unit for the selection of orientation chips for inputs from the previous layer. Multiple orientation columns are set up, each containing multiple orientation chips arranged in a specific way. The generation of orientation chip weights is conducted offline on the computer by SOM training. The update of the weight, the topological neighborhood neuron distances and the time-dependent indices are calculated as follows:

(5) $$W_{t+1}\left(r\right)=W_{t}\left(r\right)+\alpha \sigma \left(r,x\right)\left[V_{t+1}-W_{t}\left(r\right)\right]$$

(6) $$\sigma \left(r,x\right)=e^{-{∥r,x∥}^{2}}$$

(7) $$\alpha =\frac{1}{1+\frac{t}{T}}$$

where *W*, *V*, σ, α, *x*, *t*, and *T* denote the orientation chip weight, input vector, neighborhood function value, learning rate, winning node, number of iterations, and total number of iterations, respectively.

**Figure 10:**
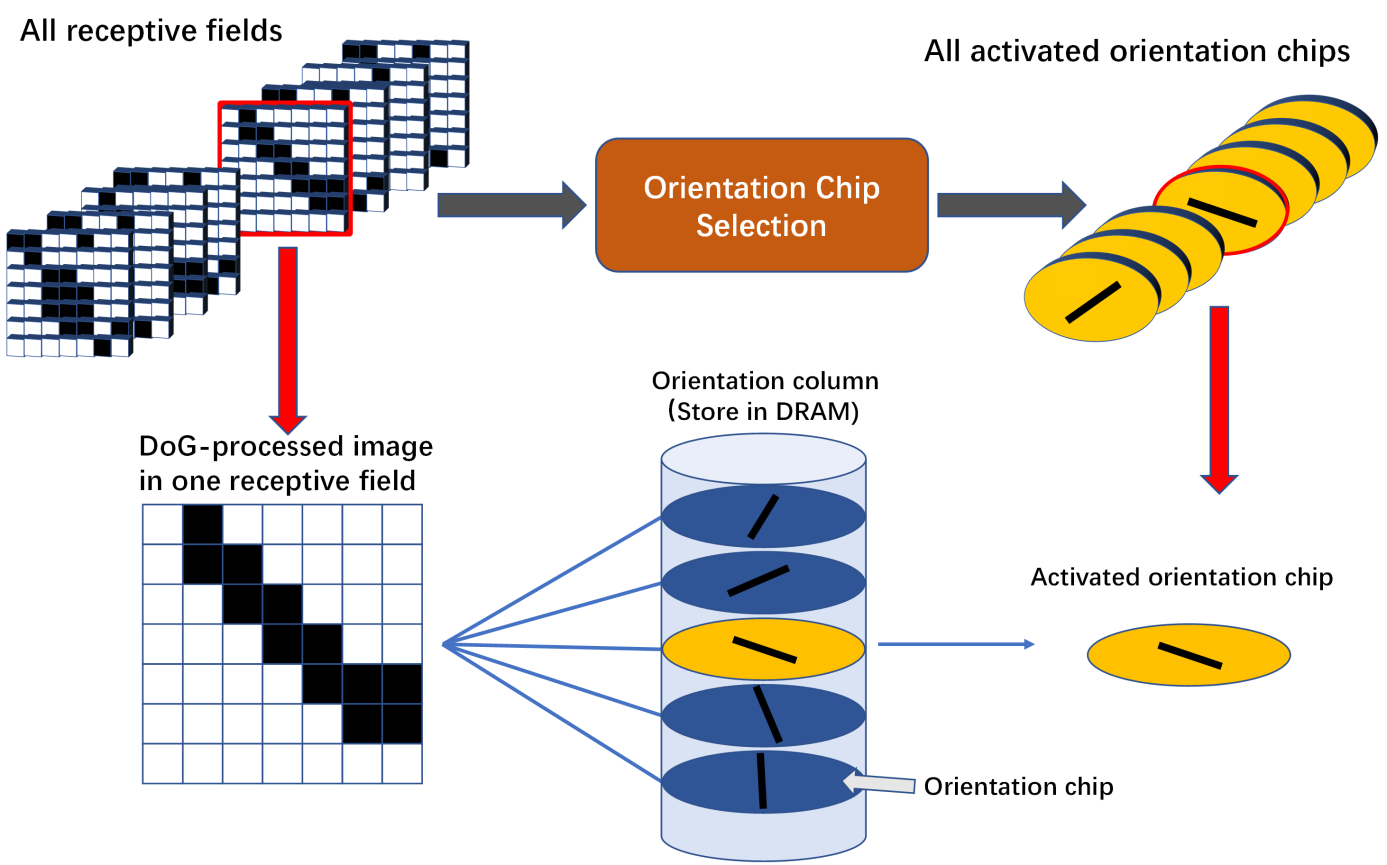
Process of selecting orientation chip. Each receptive field has a different set of orientation columns corresponding to it. The most similar orientation chip will be selected by the selection module.

**Figure 11:**
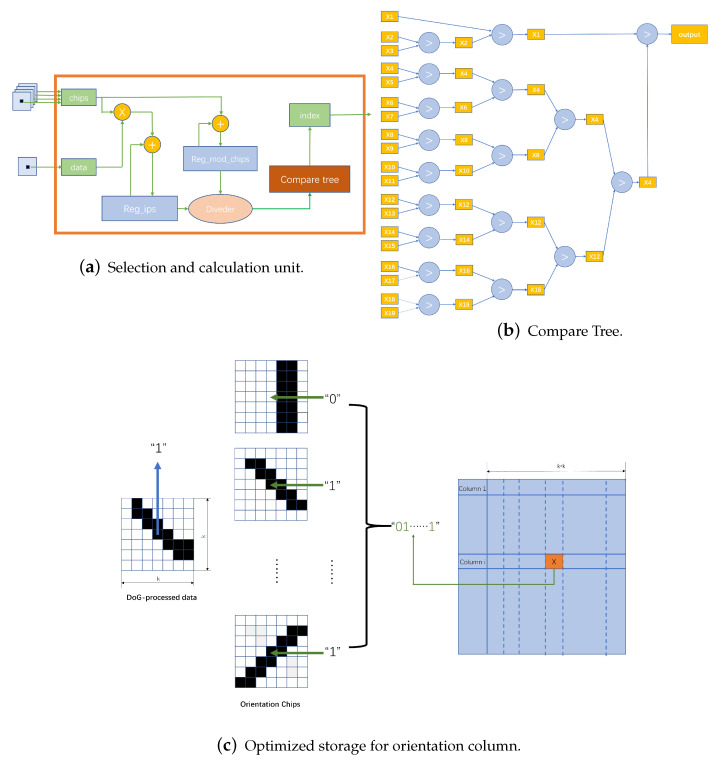
Selection module. (**a**) With the clock signal, the inner product of the vectors and the modes of the vectors of the orientation chips are calculated respectively, and after completing the calculation of the 19 orientation chips, the distance is solved by applying the division ip kernel. (**b**) The comparator takes five clock cycles to produce the result, which will always be stored in the X1 register as the output result. (**c**) X stores the decimal number of the 19 orientation chips in that position. The i-th value of the 19 orientation columns is sequential to form a binary number, which is stored in the i-th bit of the orientation column vector, corresponding to the i-th node.

The completed training orientation chip weights are stored in DRAM for orientation chip selection. When the pixels in the receptive field are processed by the DOG block, the distance selection calculation is performed with the pre-trained orientation chip array. The training process can be performed quickly using only the CPU. As shown in Figure 10, all the orientation chips in the same orientation column perform vector distance calculations. The most responsive orientation chip will be selected according to the selection algorithm and its index will be stored. The selection of the calculation block is shown in Figure 11a, which is designed by using a finite state machine approach and decomposing the calculation block into three steps: accumulation, division, and comparison. The distance is calculated by choosing Euclidean distance or cosine similarity. In the distance comparison of multiple orientation chips, the comparison can be accelerated with a comparator tree as shown in Figure 11b.

#### 4.2.4. Orientation Column Storage Optimization

In the process of orientation chip selection, all GCs in the same receptive field are connected to multiple orientation chips one by one, i.e., the GCs are calculated and compared with 19 orientation chips, which makes the reading time delay much higher than the calculation time delay, thus reducing the overall speed of the system. The storage of the orientation column weights can now be optimized by using a binary storage method.

As shown in Figure 11c, the data within a certain receptive field are processed to compare the distances orientation chips, and we use only one row of vectors to store the information of the whole orientation column. Its length is the number of GCs in the same receptive field, and its bit width is the number of orientation chips under the same orientation column.

The calculation of orientation chip selection is converted into a binary calculation, and the intermediate results of one position are calculated at the same time, which can greatly speed up the selection of the orientation chip.

## 5. Results

We program for the model by using Verilog Hardware Description Language (HDL) and simulate it by Vivado and Modelsim. The input of the image is an 8-bit grayscale image of 123 ∗ 183. It is a snapshot of the input image and belongs to the receptive field layer. Eventually, we can obtain the sequence numbers of the activated orientation chips from output.

### 5.1. Simulation for Model

**Figure 12:**
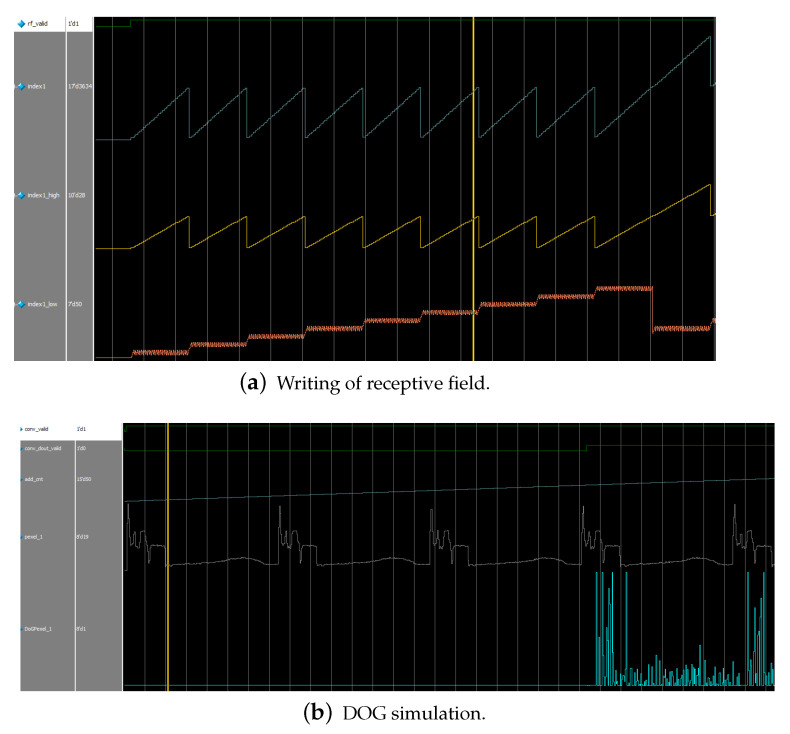
Retina cell simulation. (**a**) The Index1_high represents the No. of the receptive field being processed and the index1_low represents the position being written in the receptive field. Each small jump is a read of a particular row of indexes, and a large jump represents the completion of reading a particular row of the receptive field array. (**b**) Due to the line cache setting, it takes 3 ∗ width clock periods for the read pixel value (pexel_1) to generate the convolution result.

As shown in Figure 12a, with the change of the receptive field write signal (rf_valid), the read of the index block random access memory (BRAM) is performed, and each clock reads the four indexes corresponding to the current pixel at the same time.

Only the first index corresponding to each pixel is shown, indicated by the blue line (index1). By the time the maximum jump is generated, the writing process of a receptive field is completed. The image to be processed is read simultaneously with the index counter, and the pixel values are obtained and written to the receptive field DRAM array.

When it comes to the DOG processing stage, with the validity of the conv_valid signal, the read and convolution operations are performed on a certain receptive field with the self-increasing address signal as shown in Figure 12b. With the change of the conv_dout_valid signal, the intermediate result of the processing (DoG pixel) is transferred to the storage of the GC DRAM array.

**Figure 13:**
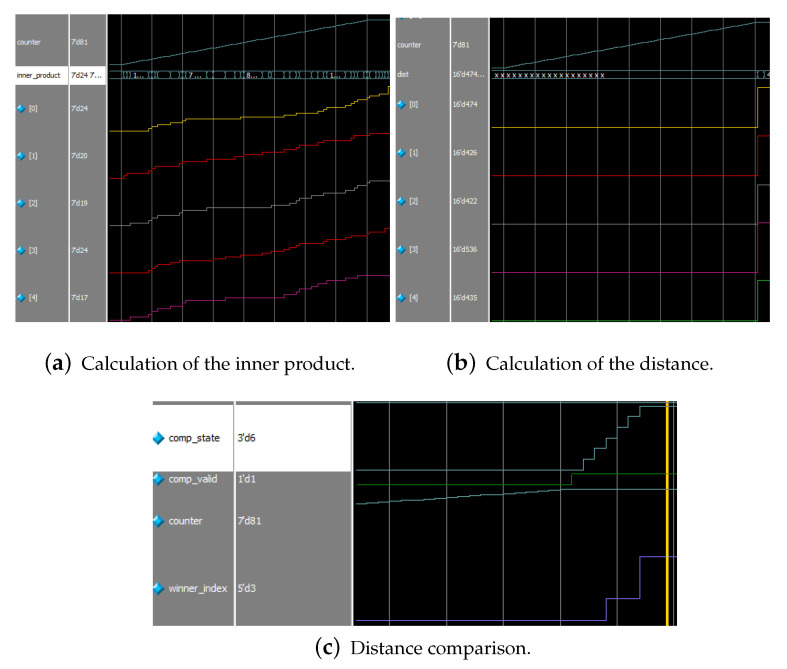
Three stages of calculation.

Finally, the simulation will enter the orientation chip selection phase, where the trained weights are expanded in bitwise form for distance calculation and comparison with the intermediate calculation results. The orientation chip selection module performs the computation after the select_valid signal is valid. Figure 13a,b show the parallel computation of products of a set of orientation chips, with the distance computation performed after 81 (the number of GC cells in one receptive field) cycles. Figure 13c illustrates that the distance comparison is performed for five clock cycles when the cmp_valid signal is valid, and the optimal orientation chip sequence number is output after completion. We mark the activated indexes in the orientation column map, thereby generating the result of the orientation column representation of the object.

### 5.2. Resource Consumption

Having shown that the visual information is correctly processed by our FPGA visual pathway model, let us look at its resource usage. With the analysis tool provided by Vivado, we can identify the resources used by the designed computational module as shown in Table 1. In our visual pathway model, information within a single receptive field is the basic unit of visual processing. Here, we show the resources needed for a single receptive field. BRAM resources are mainly used for image storage and index matrix storage, whereas DRAM is used for the receptive field most, which occupies look-up table (LUT) and flip-flop (FF) resources. Due to the conversion to binary image processing while facilitating bitwise operations, the on-chip digital signal processor (DSP) is mainly used in the distance comparison of the division calculation process and MAC of the DOG process. However, since the pixel overlap makes the DOG double computed, the resource utilization of the global-based DOG will be much lower than that of the receptive field-based DOG. This means that the selector block takes up the majority of the DSP. Depending on the resources required for a single receptive field during the whole process, we can determine the parallelism of the model based on the amount of resources.

### 5.3. Parallelism Exploration

The resource utilization of a single receptive field is given above, and the overall parallelism of the system will be constrained by the onchip resources. In terms of receptive fields, we define RFL as the delay time required from the formation of a single receptive field to the generation of activation indexes of orientation columns. Whereas the delay in generating the receptive field DRAM array comes mainly from the image signal input, segmenting and writing the receptive field is performed in real time. We simulate and synthesize based on receptive fields in parallel and 400 receptive fields in parallel with the xc7k325t chip, respectively. Our results are shown in Table 2. In the case of meeting the timing requirements, our system can run at 235.8 Mhz in the former case, while the latter can run at 222.5 Mhz. It can be found that the maximum frequency is less affected by the parallelism in our design. Table 2 shows that as the parallelism increases, the number of DSPs becomes the bottleneck first, followed by the number of LUTs bottleneck. Since DSPs are mainly used on the division of the parallel division of orientation chips, for chips with fewer computational resources, it may be necessary to reduce the division accuracy and extend the division cycle, which will add several cycles of latency.

We then take chip xc7k480t, which own more resources, to accomplish 600 receptive fields in parallel. This is a degree of parallelism that allows the parallel processing of all the receptive fields of the test images (123 × 183). Base on this parallelism, we can complete the scanning process of the images, which corresponds to the receptive field layer of our hierarchical network model. We also test all parallelism on this chip. As shown in Table 3, it not only achieves a higher degree of parallelism compared to the xc7k325t, but also gains about 3% frequency improvement. At the same time, compared to the previous chip, the parallelism has less impact on the maximum clock frequency, thanks to its resource size.

### 5.4. Comparison with CPU

We put this visual pathway processing flow on the CPU for testing. On the CPU side, we choose AMD 4800H (8 cores and 16 threads) for test by Python. For the processing latency of a single receptive field, we obtain the processing results on the CPU and FPGA, respectively, as shown in Table 4. This shows that we reduce the latency on FPGA by about 4200 times. We set the throughput as the number of receptive fields that can be processed in one second. To speed up the CPU processing, we transform the receptive field data into matrix form. Then we process the same number of receptive fields as the test image on the CPU and set the parallelism to 600. Every clock cycle on the FPGA will get an active index of the orientation columns. Table 4 shows that in terms of throughput, we also achieve a speedup ratio of 3600 times. Also in terms of power consumption, there is no doubt that FPGAs gain a huge advantage. The parallel computation on FPGAs is truly parallel in the sense that it simulates the neuro-visual mechanism very well. For our orientation selection model, both the longitudinal processing of visual pathways and the lateral processing of multiple pathways in parallel are substantially improved.

From the previous experiments, our model can generate cortical orientation maps with a high degree of similarity and orientation selectivity for information within the receptive field. Through the simulation and synthesis experiments, we can obtain that the system has low latency with good real-time performance, and the overall power of the system is low. If it is used in the visual aid system, it can obtain better endurance.

### 5.5. Comparison with GPU

We also set the visual pathway model to experiment on the GPU (RTX 3090Ti). From Table 5, we can see that when dealing with the single receptive field, the latency is reduced compared to the CPU but there is still distance from the FPGA implementation. As a part of the computer system, the GPU still needs to interact with the computer CPU, etc., which leads to a high latency. For portable wearable devices, low latency and good real-time performance are necessary requirements. FPGAs have a great advantage in this regard.

In terms of throughput, although it can be further improved with GPU, there is a huge increase in power consumption. The power consumption of the graphics card alone reaches 450 W and requires additional heat dissipation. The GPU cannot run on its own and needs to be run on the computer, which also needs to consider the overall power consumption. There is no doubt that FPGAs achieve a higher power consumption ratio than GPU. At the same time, due to the size of the computer itself, they do not have portability but also do not meet our original intention of designing a brain-like application system.

### 5.6. Orientation Chip Training Performance

The training approach proposed in this paper differs slightly from traditional SOMs due to the incorporation of connectivity optimization and topological structure optimization. However, within a single receptive field, its connection scheme shares similarities with conventional SOMs, making it practically significant when compared to other FPGA-based SOM training studies. As shown in Table 6, the experimental results in this paper are compared with the findings of a prior study [39], revealing that our approach achieves higher CUPS (computations per second). This improvement in performance is attributed to the optimized storage of the proposed approach and the rational design of numerical representation and fixed-point decimal arithmetic modules, resulting in reduced hardware resource utilization, such as LUTs. One major advantage of our approach is its capability to handle training on larger-scale neural layers, thereby achieving higher CUPS. However, the high dimensionality of the input layer in this paper leads to significant delays in weight IO operations, resulting in a slight reduction in the maximum clock frequency. Nevertheless, this trade-off leads to a substantial overall improvement in CUPS.

Our proposed training approach is compared with other algorithms as shown in Table 7. The table presents the performance of training with a single orientation column (row 3) and dual orientation columns (row 6) when the neuron count is similar. Our training system achieves higher CUPS compared to the first two systems when training with a single orientation column. However, there is still a gap compared to the results of [40]. We optimize the connections between the input layer and the competition layer, removing unnecessary connections that can be considered connections with update magnitudes of 0. Similar connection optimizations have been used in other studies [41] to improve CUPS. Considering the number of connections, our approach can further enhance CUPS when training with dual orientation columns simultaneously.

### 5.7. Representation Experiment of the Visual Pathway Model

**Figure 14:**
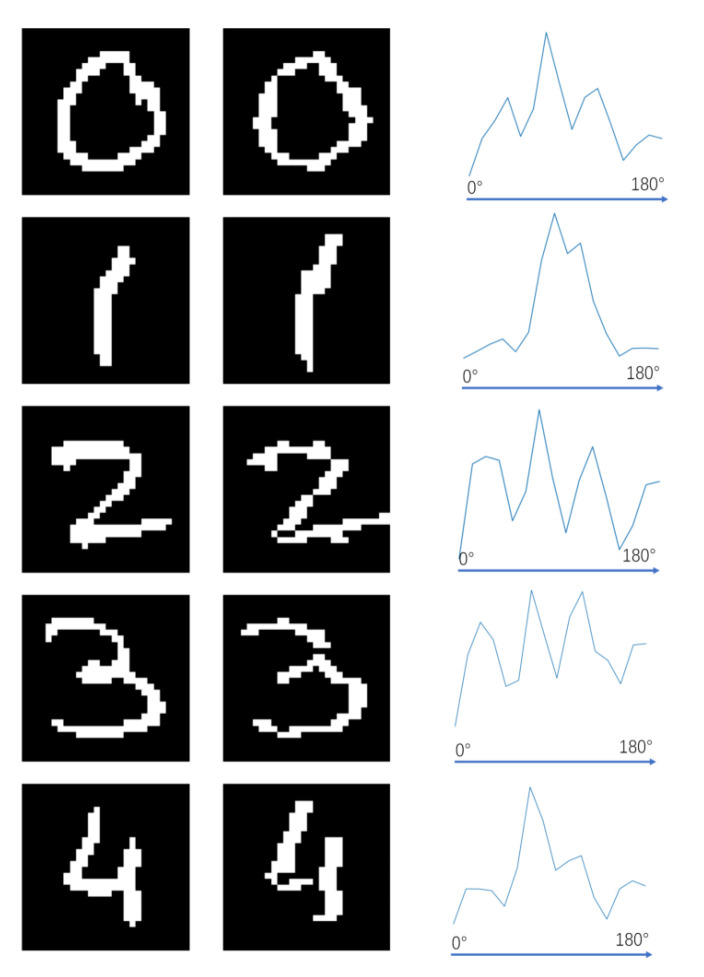
The first column shows the original image from the Mnist dataset, the second column displays the image restored using the orientation chips array representation, and the third column represent the various orientations of the orientation patches activated by the same digit, with the x-axis ranging from 0 to 180 degrees.

The representation results of the orientation chips array are examined at two different scales. Firstly, we observe the representation results of the orientation chips array on images with a smaller resolution, such as the Mnist dataset, which has a resolution of 28 × 28 pixels. The array should be able to extract line segments of various orientations for different digits. By using orientation chips instead of line segments, the array should be capable of reconstructing the original image. The specific experimental results are shown in Figure 14. As seen in the second column of the figure, the final representation results can effectively reconstruct the original digits for different numbers. This indicates that the GCs array trained in this study can represent and reconstruct images.

We conduct a statistical analysis of the activated orientation patch types for each digit, which provides the proportions of different orientation patches activated by different digits as shown in the third column. From the graph, it can be observed that for digit 0, the activation of various types of orientation chips is evenly distributed. This can be attributed to the circular structure of the digit. As the digit itself has a slender and tall shape, there are more orientation chips biased towards the vertical direction compared to the horizontal direction. On the other hand, for digit 1, a few specific types of orientation chips are prominently activated, with their optimal orientations mostly close to the vertical direction. This is likely due to the prevalence of inclined angles in handwritten characters, resulting in a higher activation of orientation chips biased towards the vertical direction. As for digits 2 and 3, although their shapes are somewhat similar, digit 3 exhibits a higher activation of orientation chips along the main diagonal. The representation results of the orientation chips array indicate the effectiveness of the multi-layer array proposed in this study.

The ability to effectively represent low-resolution images is a prerequisite for achieving the successful representation of higher-resolution images. We further conduct experiments on images with higher resolutions. We capture partial images using CCD devices, and some images are obtained from the BSD dataset [45].

**Figure 15:**
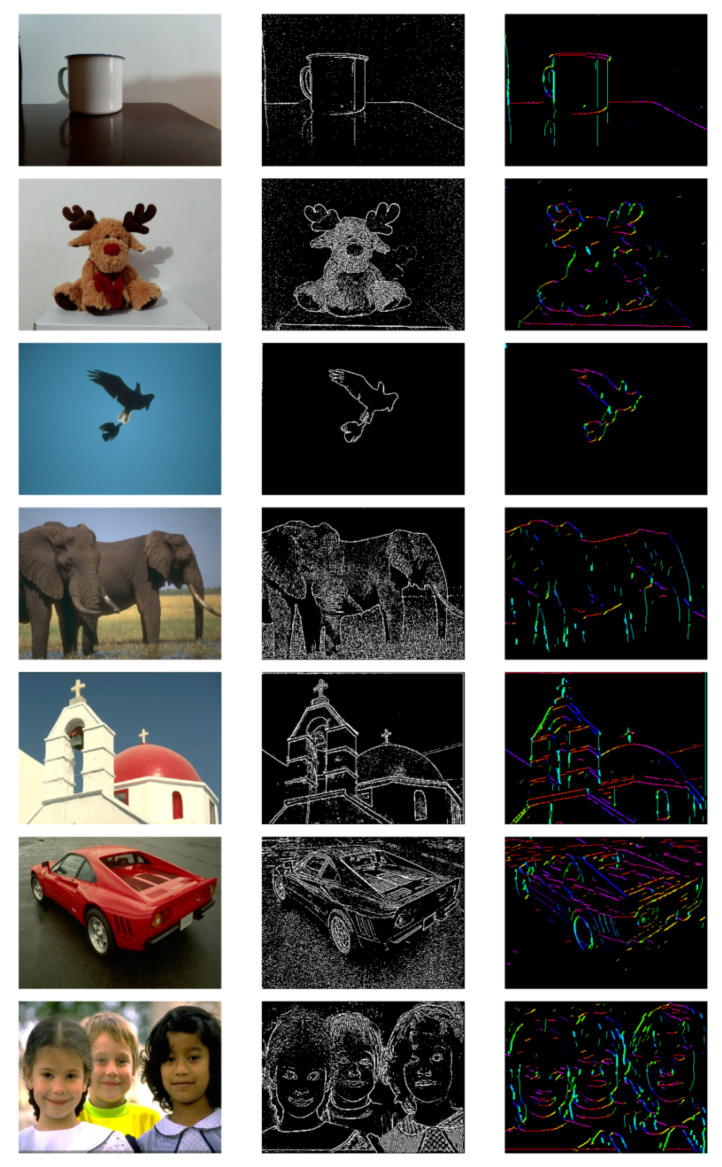
The first column shows the original image, with a portion derived from the BSD dataset and another portion captured by CCD devices, the second column shows the image after processing with the GCs receptive field array, and the third column shows the image after activation of the orientation chips array. Different colors correspond to different optimal orientations of the orientation chips, which are consistent with the colors in the artificial cortical map.

As shown in Figure 15, the array captures the variations in brightness and darkness in the edge regions, making the edges of the image more pronounced. However, the extracted results are coarse and accompanied by a significant amount of salt-and-pepper noise. The third column represents the results of the orientation chips array’s representation of the image. The entire image is composed of multiple oriented chips resembling line segments, with different colored lines representing patches with different optimal orientations. Compared to the processing results of the GCs array, the OCs array significantly reduces noise and eliminates numerous invalid edges, resulting in a clearer representation. From this representation result, we can obtain both a feature descriptor to describe the entire image and the distribution of features with the same orientation within the image.

**Figure 16:**
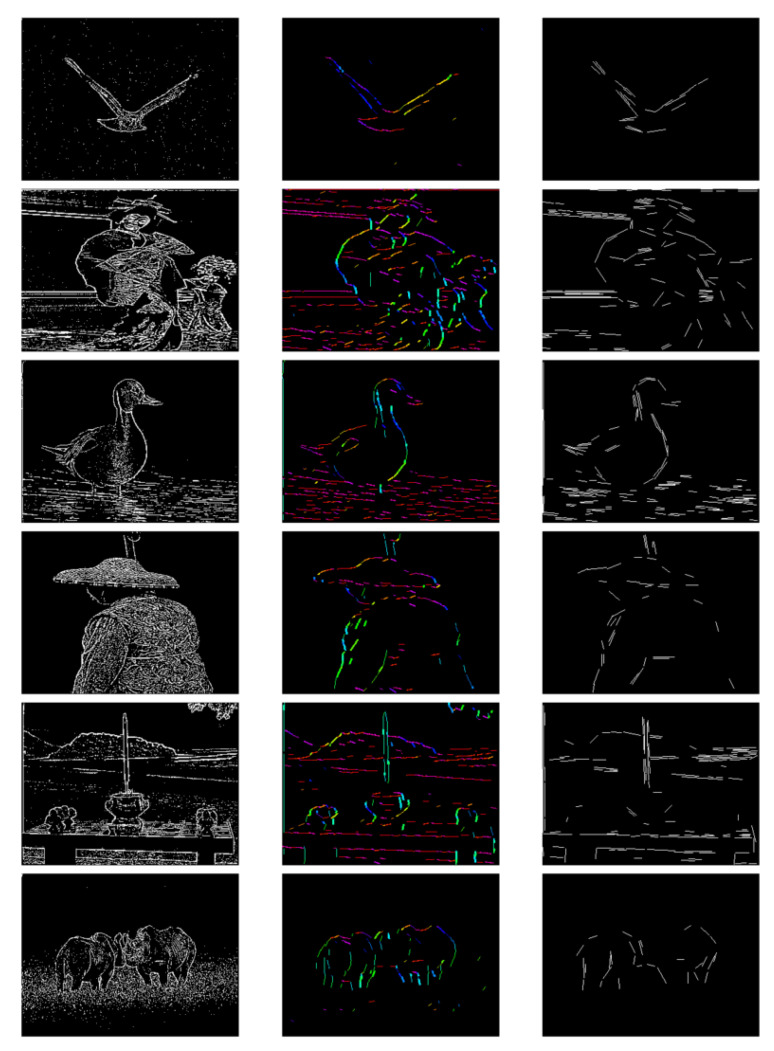
The first column represents the results obtained after processing with the GCs receptive field array. The second column displays the results after activation of the OCs array, and the third column presents the results obtained through the LSD algorithm. All the original images are sourced from the BSD dataset.

Based on the results obtained after processing with the GCs array, we compare the capability of extracting and representing orientation information with the LSD algorithm [46] using the orientation columns array. As shown in Figure 16, compared with the LSD method, for relatively simple graphic features, such as the eagle in the third row, the training results can better reflect the representation ability of the visual functional column for edge orientation. For images containing complex information, such as an image with three people, the model focuses more on extracting the excessively redundant features, identifying key lines as a prerequisite, and then distinguishing the orientation. It can be observed that the orientation column array extracts orientation line segments that exhibit more continuity and form closed contours; one reason for this is that the model has a smaller receptive field, allowing for more detailed representation. Furthermore, it can be observed that within the same region, there are no multiple line segments of similar length with different orientations. This indicates that the training of the orientation columns can better learn the features of natural images and activate orientation chips that best match the edge information in the images.

## 6. Discussion and Conclusions

In this study, we propose a physiologically consistent hierarchical network model of the primary visual cortex orientation selection, which is bionic and highly parallelizable. The network dissects the physiological basis of orientation selection and generates highly approximate maps of cortical orientation columns.

Then we map the network model hierarchy on FPGAs and simulate the orientation selection of objects and implement it. The method achieves the integration of storage and computation and realizes the functional decomposition and fine-grained mapping of multi-level neuronal network computational architectures to FPGA functional components.

As [47] indicates, based on the neutron structure, brain-like chips can overcome the von Neumann limitation and improve both the speed and complexity of the calculation. The power consumption will decrease at the same time. Our FPGA design can dramatically speed up the visual pathway processing speed and increase the parallelism. The low-latency, low-power, and high-parallelism characteristics of the model are well suited for building assistive systems to help the visually impaired. Simulating the signal processing in the biological cortex makes the input and output of the computing system biologically interpretable and compatible with the interface protocol, which is helpful for the brain–computer interface connection.

In the future, we will conduct larger-scale biocomputing and explore higher-level visual models in other visual cortices and embedded wearable devices to help solve more visual-impairment problems.

## Figures and Tables

**Table 1:** Resource consumption of visual pathway.

	LUTs	FFs	BRAM	DSP
Index Matrix	40	8	11	0
FIFO	110	63	0	0
MAC	145	26	0	2
Receptive Field	16	8	0	0
Ganglion Cell	16	8	0	0
weight	28	7	0	0
Selector	336	461	0.5	2

**Table 2:** Synthesis results on xc7k325t.

Parallelism	100	400
LUT(203800)	21.1%	88.7%
FF(407600)	13.6%	55.9%
BRAM(445)	22.7%	56.4%
DSP(840)	24.5%	95.5%
Fmax	235.8 Mhz	222.5 Mhz
RFL	483 ns	520 ns
Power	1.61 W	5.34 W

**Table 3:** Synthesis results on xc7k480t.

Parallelism	100	400	600
LUT(298600)	14.4%	57.7%	88.74%
FF(597200)	8.6%	34.4%	51.6%
BRAM(955)	10.5%	26.2%	36.8%
DSP(1920)	24.5%	41.7%	62.6%
Fmax	241.0 Mhz	238.1 Mhz	236.4 Mhz
RFL	474 ns	479 ns	482 ns
Power	1.70 W	5.53 W	8.06 W

**Table 4:** Comparison results with CPU.

Platform	Latency (ns)	Throughput (k)	Power (W)
CPU(AMD 4800H)	2,030,372	61	45
FPGA(xc7k480t)	484	202,167	8

**Table 5:** Comparison results with GPU.

Platform	Latency (ns)	Throughput (k)	Power (W)
GPU(RTX 3090Ti)	151,157	335,432	450
FPGA(xc7k480t)	484	202,167	8

**Table 6:** Hardware resource utilization in SOM training.

	Neurons	Input Dimension	Connections	LUT	Fmax (MHz)	CUPS
Our model	19	81	1539	3224	1.05	1613
[39]	25	3	75	8845	1.51	113

**Table 7:** Performance comparison of CUPS.

	Neurons	CUPS
[42]	49	300
[43]	32	853
Single orientation column	19	1613
[44]	36	4200
[40]	25	5200
Dual orientation columns	38	6463

## Data Availability

No dataset is acquired in this research.

## List of Abbreviations

FPGA	field programmable gate array
LGN	lateral geniculate nucleus
GCs	ganglion cells
CPU	central processing unit
GPU	graphics processing unit
DOG	difference of Gaussians
On-P	on-center parvo cells
Off-P	off-center parvo cells
SOM	self-organizing map
FIFO	first input first output
MAC	multiple access channel
DRAM	dynamic random access memory
HDL	hardware description language
BRAM	block random access memory
FF	flip flop
DSP	digital signal processor
LUT	look-up table

## References

[1] Wu W.H., Bui A.A., Batalin M.A., Au L.K., Binney J.D., Kaiser W.J. (2008). MEDIC: Medical embedded device for individualized care. Artif. Intell. Med..

[2] Papernot N., McDaniel P., Jha S., Fredrikson M., Celik Z.B., Swami A. The limitations of deep learning in adversarial settings. Proceedings of the 2016 IEEE European Symposium on Security and Privacy (EuroS&P).

[3] Goodfellow I.J., Shlens J., Szegedy C. (2014). Explaining and harnessing adversarial examples. arXiv.

[4] Anthony L.F.W., Kanding B., Selvan R. (2020). Carbontracker: Tracking and predicting the carbon footprint of training deep learning models. arXiv.

[5] Strubell E., Ganesh A., McCallum A. (2019). Energy and policy considerations for deep learning in NLP. arXiv.

[6] Salamat S., Imani M., Khaleghi B., Rosing T. F5-hd: Fast flexible fpga-based framework for refreshing hyperdimensional computing. Proceedings of the 2019 ACM/SIGDA International Symposium on Field-Programmable Gate Arrays.

[7] Wei H., Wang X.M., Lai L.L. (2011). Compact image representation model based on both nCRF and reverse control mechanisms. IEEE Trans. Neural Netw. Learn. Syst..

[8] Wei H., Lang B., Zuo Q. (2013). Contour detection model with multi-scale integration based on non-classical receptive field. Neurocomputing.

[9] Wei H., Li H. (2014). Shape description and recognition method inspired by the primary visual cortex. Cogn. Comput..

[10] Wei H., Li Q., Dong Z. (2014). Learning and representing object shape through an array of orientation columns. IEEE Trans. Neural Netw. Learn. Syst..

[11] Tanaka Y., Tamukoh H. Hardware implementation of brain-inspired amygdala model. Proceedings of the 2019 IEEE International Symposium on Circuits and Systems (ISCAS).

[12] Tanaka Y., Morie T., Tamukoh H. (2020). An Amygdala-Inspired Classical Conditioning Model Implemented on an FPGA for Home Service Robots. IEEE Access.

[13] Aggarwal A. (2016). Neuromorphic VLSI realization of the hippocampal formation. Neural Netw..

[14] Cho Y.C.P., Bae S., Jin Y., Irick K.M., Narayanan V. Exploring Gabor filter implementations for visual cortex modeling on FPGA. Proceedings of the 2011 21st International Conference on Field Programmable Logic and Applications.

[15] Zidan M.A., Strachan J.P., Lu W.D. (2018). The future of electronics based on memristive systems. Nat. Electron..

[16] Guo K., Sui L., Qiu J., Yu J., Wang J., Yao S., Han S., Wang Y., Yang H. (2017). Angel-eye: A complete design flow for mapping cnn onto embedded fpga. IEEE Trans. Comput. Aided Des. Integr. Circuits Syst..

[17] Zhang C., Wu D., Sun J., Sun G., Luo G., Cong J. Energy-efficient CNN implementation on a deeply pipelined FPGA cluster. Proceedings of the 2016 International Symposium on Low Power Electronics and Design.

[18] Rice K.L., Bhuiyan M.A., Taha T.M., Vutsinas C.N., Smith M.C. FPGA Implementation of Izhikevich Spiking Neural Networks for Character Recognition. Proceedings of the 2009 International Conference on Reconfigurable Computing and FPGAs.

[19] Pearson M.J., Pipe A.G., Mitchinson B., Gurney K., Melhuish C., Gilhespy I., Nibouche M. (2007). Implementing Spiking Neural Networks for Real-Time Signal-Processing and Control Applications: A Model-Validated FPGA Approach. IEEE Trans. Neural Netw..

[20] Khodamoradi A., Denolf K., Kastner R. S2N2: A FPGA Accelerator for Streaming Spiking Neural Networks. Proceedings of the The 2021 ACM/SIGDA International Symposium on Field-Programmable Gate Arrays.

[21] Shimonomura K., Kushima T., Yagi T. Neuromorphic binocular vision system for real-time disparity estimation. Proceedings of the 2007 IEEE International Conference on Robotics and Automation.

[22] Shimonomura K., Yagi T. (2008). Neuromorphic VLSI vision system for real-time texture segregation. Neural Netw..

[23] Delbrück T., Liu S.C. (2004). A silicon early visual system as a model animal. Vis. Res..

[24] Przybyszewski A.W., Linsay P.S., Gaudiano P., Wilson C.M. (2007). Basic Difference Between Brain and Computer: Integration of Asynchronous Processes Implemented as Hardware Model of the Retina. IEEE Trans. Neural Netw..

[25] Chen J.C., Chen R.D. (2002). Toward an evolvable neuromolecular hardware: A hardware design for a multilevel artificial brain with digital circuits. Neurocomputing.

[26] Yuan D., Manduchi R. Dynamic environment exploration using a virtual white cane. Proceedings of the 2005 IEEE Computer Society Conference on Computer Vision and Pattern Recognition (CVPR’05).

[27] Cardin S., Thalmann D., Vexo F. (2007). A wearable system for mobility improvement of visually impaired people. Vis. Comput..

[28] Ulrich I., Borenstein J. (2001). The GuideCane-applying mobile robot technologies to assist the visually impaired. IEEE Trans. Syst. Man Cybern. Part A Syst. Hum..

[29] Pradeep V., Medioni G., Weiland J. Robot vision for the visually impaired. Proceedings of the 2010 IEEE Computer Society Conference on Computer Vision and Pattern Recognition Workshops.

[30] Helal A., Moore S., Ramachandran B. Drishti: An integrated navigation system for visually impaired and disabled. Proceedings of the Fifth International Symposium on Wearable Computers.

[31] Hubel D.H., Wiesel T.N. (1962). Receptive fields, binocular interaction and functional architecture in the cat’s visual cortex. J. Physiol..

[32] Desimone R., Schein S.J. (1987). Visual properties of neurons in area V4 of the macaque: Sensitivity to stimulus form. J. Neurophysiol..

[33] McKeefry D., Watson J., Frackowiak R., Fong K., Zeki S. (1997). The activity in human areas V1/V2, V3, and V5 during the perception of coherent and incoherent motion. Neuroimage.

[34] Kuffler S.W. (1953). Discharge patterns and functional organization of mammalian retina. J. Neurophysiol..

[35] Rodieck R.W., Kiang N.S., Gerstein G.L. (1962). Some quantitative methods for the study of spontaneous activity of single neurons. Biophys. J..

[36] Kolb H., Fernandez E., Nelson R. Webvision: The Organization of the Retina and Visual System. https://pubmed.ncbi.nlm.nih.gov/21413389/.

[37] Ritter H., Kohonen T. (1989). Self-organizing semantic maps. Biol. Cybern..

[38] Blasdel G.G., Salama G. (1986). Voltage-sensitive dyes reveal a modular organization in monkey striate cortex. Nature.

[39] de Sousa M.A.d.A., Del-Moral-Hernandez E. An FPGA distributed implementation model for embedded SOM with on-line learning. Proceedings of the 2017 International Joint Conference on Neural Networks (IJCNN).

[40] Hikawa H., Maeda Y. (2015). Improved learning performance of hardware self-organizing map using a novel neighborhood function. IEEE Trans. Neural Netw. Learn. Syst..

[41] de Sousa M.A.d.A., Del-Moral-Hernandez E. Comparison of three FPGA architectures for embedded multidimensional categorization through Kohonen’s Self-organizing maps. Proceedings of the 2017 IEEE International Symposium on Circuits and Systems (ISCAS).

[42] Długosz R., Kolasa M., Szulc M. An FPGA implementation of the asynchronous programmable neighborhood mechanism for WTM self-organizing map. Proceedings of the 18th International Conference Mixed Design of Integrated Circuits and Systems-MIXDES 2011.

[43] Ramirez-Agundis A., Gadea-Girones R., Colom-Palero R. (2008). A hardware design of a massive-parallel, modular NN-based vector quantizer for real-time video coding. Microprocess. Microsyst..

[44] Ben Khalifa K., Blaiech A.G., Bedoui M.H. (2019). A novel hardware systolic architecture of a self-organizing map neural network. Comput. Intell. Neurosci..

[45] Martin D., Fowlkes C., Tal D., Malik J. A database of human segmented natural images and its application to evaluating segmentation algorithms and measuring ecological statistics. Proceedings of the Eighth IEEE International Conference on Computer Vision (ICCV).

[46] Von Gioi R.G., Jakubowicz J., Morel J.M., Randall G. (2012). LSD: A line segment detector. Image Process. Line.

[47] Talha M. (2020). A history of development in brain chips in present and future. Int. J. Psychosoc. Rehabil..

